# Analysis of Cochrane systematic reviews: A comprehensive study of impact and influence from 1998 to 2024

**DOI:** 10.1002/cesm.70010

**Published:** 2024-12-09

**Authors:** Amin Sharifan

**Affiliations:** ^1^ Department for Evidence‐based Medicine and Evaluation University for Continuing Education Krems Krems an der Donau Austria

**Keywords:** bibliometrics, cochrane, developed countries, developing countries, forecasting, sexual and gender minorities, systematic reviews

## Abstract

**Introduction:**

Evidence from Cochrane systematic reviews has significantly impacted clinical practices across diverse disciplines and is widely integrated into international guidelines. To date, there are no bibliometric analyses of Cochrane's publications.

**Methods:**

The search encompassed the Scopus database from inception to May 2024, with results limited to studies published by the Cochrane Database of Systematic Reviews. The analysis examined annual trends and publication volumes, citation patterns, contributing countries, authors, institutions, funding sources, and common keywords. Scopus' built‐in analytical tools, the bibliometrix package in RStudio, and VOSviewer software facilitated the analysis of the results.

**Results:**

A total of 12,150 systematic reviews were eligible. There was a fluctuating pattern in publication and citation trends within Cochrane reviews, with a decline in both metrics since 2016. Contributions mainly came from high‐income countries, their institutions, and authors residing there, with significant government funding supporting publications in these regions. The United Kingdom (27%), Australia (10%), and the United States (9%) had the greatest contributions among other countries, respectively. Furthermore, the demographic emphasis in Cochrane reviews was concerned with female and male participants as well as children and adult populations, hinting at the potential underrepresentation of minor gender identities and older adults in the synthesis of evidence.

**Conclusion:**

Cochrane should actively involve researchers and experts from low‐ and middle‐income countries in evidence synthesis, ensure underrepresented and low‐resource regions are included in its emerging Evidence Synthesis Units and Thematic Groups, and prioritize the inclusion of geriatric populations and sexual and gender minorities in its evidence to enhance inclusivity and global representation.

## 1 INTRODUCTION

Well‐conducted systematic reviews and meta‐analyses are widely regarded as having the highest quality in study designs in the hierarchy of evidence. The primary aim of systematic reviews is often to provide a conclusive answer to a clinical query by synthesizing evidence from a diverse array of independent studies, both peer‐reviewed and preprints, that share similar aspects in terms of population(s), intervention(s), comparison(s), outcome(s) of interest, and study design. Systematic reviews can address a wide spectrum of research questions using both quantitative and qualitative synthesis that range from diagnosis and treatment to prognosis, education, and quality improvement initiatives.

It is crucial to differentiate systematic reviews from narrative reviews, which primarily seek to summarize previously published literature on a specific topic and are subject to “cherry picking” or the fallacy of incomplete evidence. Narrative reviews often lack the systematic methodologies employed in systematic reviews, such as predetermined criteria for study eligibility, comprehensive search strategies, rigorous assessment of study quality and potential biases, and, in some cases, statistical analysis. This distinction is significant, as the absence of these systematic approaches in narrative reviews can lead to selection bias through the omission of key studies or the disproportionate emphasis on results from studies with flawed methodologies [1]. Therefore, the rigorous and comprehensive nature of systematic reviews and meta‐analyses makes them the most reliable source of evidence for informing healthcare decisions and policy, and the preferred sources in clinical guidelines.

The Cochrane Collaboration, a leading entity in the global evidence landscape, is dedicated to advancing evidence‐based decision‐making on a global scale [2]. Through the publication of meticulously crafted systematic reviews by expert practitioners following stringent methodologies, Cochrane plays a pivotal role in shaping healthcare practices worldwide. Embracing inclusivity as a core value, Cochrane actively engages with diverse populations, transcending geographical boundaries to ensure a broad spectrum of voices contributes to the evidence‐generation process. Additionally, Cochrane's robust framework addresses conflicts of interest among contributors, underscoring the organization's commitment to transparency and ethical standards. This comprehensive approach has firmly established Cochrane as a driving force in the evidence‐based movement, facilitating the synthesis, dissemination, and application of evidence to guide healthcare policies and clinical decisions. Collaborations with esteemed healthcare institutions like the World Health Organization further amplify Cochrane's impact, fostering synergies that expedite the translation of evidence into tangible outcomes for global health.

This study presents a novel bibliometric exploration of Cochrane systematic reviews, with the aim of comprehensively analyzing contributions and trends in evidence synthesis from 1998 to 2024. By identifying patterns, influential factors, and areas for enhancement, this study intends to guide future policy decisions and strategic planning for Cochrane's governing board.

## 2 METHODS

### 2.1 Database selection and search strategy

Various databases such as Scopus, Web of Science, and PubMed index systematic reviews published by Cochrane to enhance their scholarly impact and accessibility. These reviews are also available within the Cochrane Library. The author deliberately selected the Scopus database due to its accessibility, advanced visualization tools, and robust functionality for conducting detailed citation analyses. The search query was broad so it can retrieve all articles published by Cochrane up to May 17, 2024. The searching process involved applying a search within the source title filter of the database using the key term “Cochrane.” The search included no restrictions on language, publication date, document type, source type (e.g., journals or books), or publication stage of the articles.

### 2.2 Criteria for identifying and retaining articles

Upon executing the search query, the author exported the results from the Scopus database in a raw comma‐separated value file format and Microsoft Excel Version 2108 facilitated the deduplication of these results based on their digital object identifier. Initially, the author verified that the articles had identical titles, abstract, and authors, and then summed the citation counts from both entry data and retained the record with more comprehensive information, for instance, in terms of keywords and funding sources.

### 2.3 Data analysis

To validate the relevance of the retrieved articles, the author manually reviewed a random sample of the titles and abstracts to confirm their alignment with the scope and objectives of the current investigation. Subsequently, the acquired data underwent a comprehensive analysis and visualization, utilizing Scopus' built‐in analyzer, the bibliometrix package version 4.1.4 via RStudio version 2023.12.0 [3], and the VOSviewer software version 1.6.20 (Leiden University, Leiden, Netherlands) in the same file format. This evaluation encompassed a systematic examination of publication trends, authorship patterns, country‐specific productivity, funding sources, keyword frequencies, and citation analysis.

## 3 RESULTS

**Figure 1:**
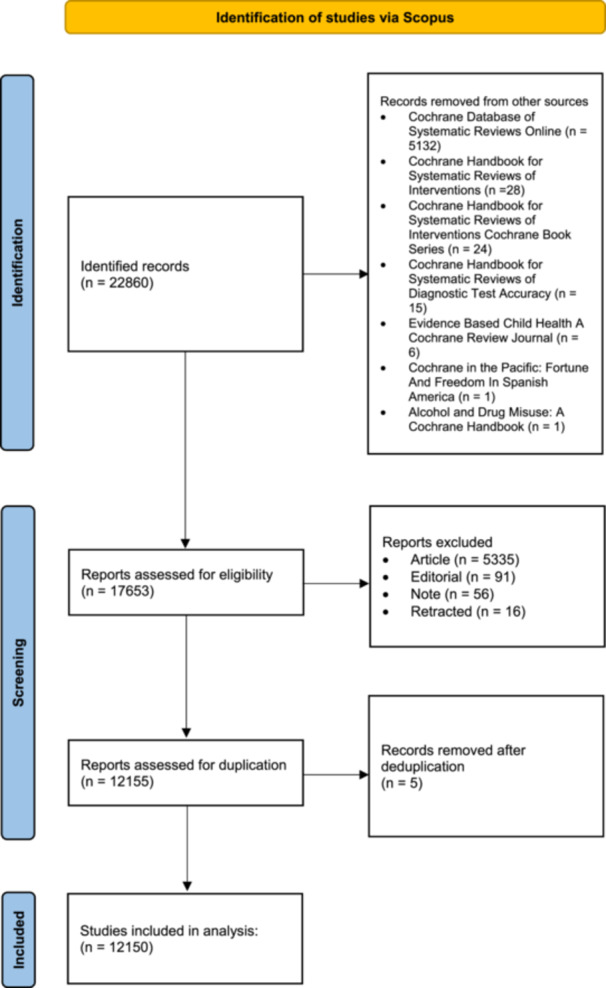
Study selection process.

The initial search yielded 22,860 articles. Manual screening of titles revealed identical titles in studies published under the Cochrane Database of Systematic Reviews and Cochrane Database of Systematic Reviews Online, with the latter lacking digital object identifiers. Considering that updating outdated reviews in light of new data is common in evidence‐based medicine, and updated reviews often retain identical or highly similar titles to their previous versions, deduplication based solely on the titles risked reducing the number of eligible results and potentially excluding important updated reviews with valuable findings. To mitigate this risk and manage the large volume of studies, the author limited the eligible studies to those published in the Cochrane Database of Systematic Reviews source, reducing the total number of results to 17,653 studies. Further manual examination of the results showed that articles published under the document type “Article” were Cochrane protocols. Consequently, the analysis excluded protocols, as well as editorials, notes, and withdrawn articles, as they did not contain any original data synthesis (Figure 1).

### 3.1 Publication trends and impact

**Figure 2:**
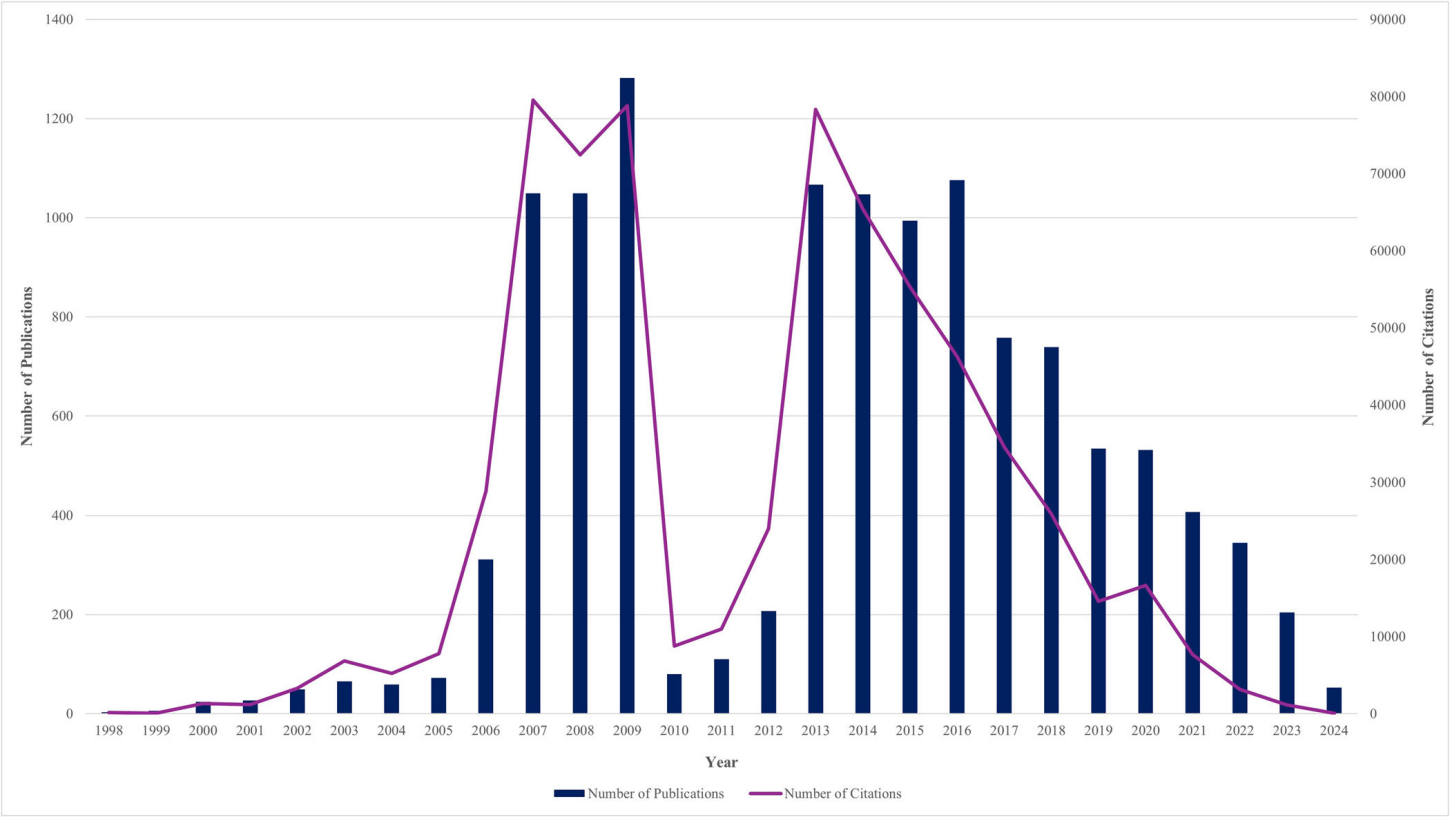
Trends in Cochrane review publications and citations indexed in Scopus (1998–2024). The blue columns represent the number of publications (left *y*‐axis), while the pink line indicates the number of citations (right *y*‐axis).

Between 1998 and 2024, Cochrane published a total of 12,150 review articles, with an average annual growth rate of 11.68%. The publication trend exhibited significant fluctuations, with a steady increase from 1998 to 2009, peaking at 1282 publications in 2009, followed by a sharp decline to 80 reviews in 2010. Subsequently, there was a fluctuating pattern with an increase in publications between 2010 and 2016, reaching 1076 in 2016, albeit with a minor decrease in 2015. However, since 2016, there has been a notable decline in Cochrane's evidence production, with only 53 reviews published as of May 2024, marking a 95% decrease since then. The impact of Cochrane reviews, as measured by citations, varied across years, with 1999 and 2024 receiving the least citations, and 2007, 2009, and 2013 garnering the most. Notably, evidence from 2012 had the highest impact based on the citation‐to‐publication ratio. Despite an increase in publications in 2016, the number of citations for Cochrane reviews has been on a declining trend since 2013, indicating a shift in both publication output and impact over the years (Figure 2).

### 3.2 Authorship and institutional contributions

During the 26‐year study period from 1998 to 2024, a total of 22,929 authors contributed to the production of Cochrane reviews. International collaboration was a significant factor, with 42.78% of articles involving co‐authors from different countries. On average, each Cochrane review had 4.86 co‐authors. An analysis of the top fifteen most prolific authors, ranked by their total number of published Cochrane reviews (Table 1), revealed some interesting geographic trends. Researchers from the United Kingdom were the most represented and had the most impact in terms of total citations among the top contributors, followed by authors from Australia, New Zealand, and China. However, researchers from other regions were not part of this list.

**Table 1:** Top 15 prolific authors of Cochrane reviews (1998–2024).

Author's name	Number of publications	Total citations	Primary affiliation in Scopus
Kurinchi Selvan Gurusamy	159	6671	University College London, United Kingdom
Robert Andrew Moore	155	9475	Newton Ferrers, United Kingdom
Sheena Derry	151	8809	University of Oxford Medical Sciences Division, United Kingdom
Brian R. Davidson	127	5231	University College London, United Kingdom
Taixiang Wu	92	2636	West China School of Medicine/West China Hospital of Sichuan University, China
Caroline A. Crowther	91	5503	The University of Auckland, New Zealand
Jonathan C. Craig	87	5471	Flinders University, Australia
Philip J. Wiffen	87	7835	Thame, United Kingdom
Anthony G. Marson	86	2214	University of Liverpool, United Kingdom
Christian Gluud	83	4622	Syddansk Universitet, Denmark
Philippa F. Middleton	83	6299	South Australian Health and Medical Research Institute, Australia
Andrew Bryant	79	3639	University of Newcastle upon Tyne, United Kingdom
Cindy M. Farquhar	78	7062	The University of Auckland, New Zealand
Zbys Fedorowicz	75	2070	Veritas Health Sciences Consultancy, United Kingdom
Helen V. Worthington	73	8425	University of Manchester, United Kingdom

*Note*: Of note, the ranking is determined by the authors' total number of Cochrane reviews indexed in Scopus.

Furthermore, the top 10 corresponding authors with the greatest number of publications were mostly from Western Europe, with the United Kingdom (*n* = 3819) on top of the chart, followed by the Netherlands (*n* = 544), Germany (*n* = 396), Italy (*n* = 329), and Ireland (*n* = 231). Australia (*n* = 1316) and China (*n* = 643) were the only representatives of the Oceania and Asia regions, respectively, and the same applied to Brazil from South America (*n* = 323). Corresponding authors from North America were also ranked 2nd and 3rd on this chart with 790 and 780 articles, respectively.

When analyzing the top 15 institutions with the greatest number of published Cochrane reviews, five were from the United Kingdom, four were from Australia, two were from China, and New Zealand, Canada, and the Netherlands each had one representative. In other words, except for Western Europe, Oceania, and East Asia, no institutions from other regions of the world were among the most contributors in terms of publication of Cochrane reviews (Table 2).

**Table 2:** Top 15 institutions with the greatest number of published Cochrane reviews (1998–2024).

Institution	Number of publications	Country
University of Oxford	1279	United Kingdom
University of Liverpool	477	United Kingdom
The University of Sydney	390	Australia
Sichuan University	365	China
University of Nottingham	359	United Kingdom
University of Auckland	338	New Zealand
West China School of Medicines/West China Hospital of Sichuan University	316	China
University College London	297	United Kingdom
University of Toronto	275	Canada
Amsterdam UMC ‐ University of Amsterdam	263	Netherlands
University of Adelaide	260	Australia
Monash University	243	Australia
University of Manchester	225	United Kingdom
The University of Queensland	222	Australia
University of Melbourne	216	Australia

*Note*: Of note, the ranking is determined by the institutions' total number of Cochrane reviews indexed in Scopus.

### 3.3 Countries contributions and impact analysis

**Figure 3:**
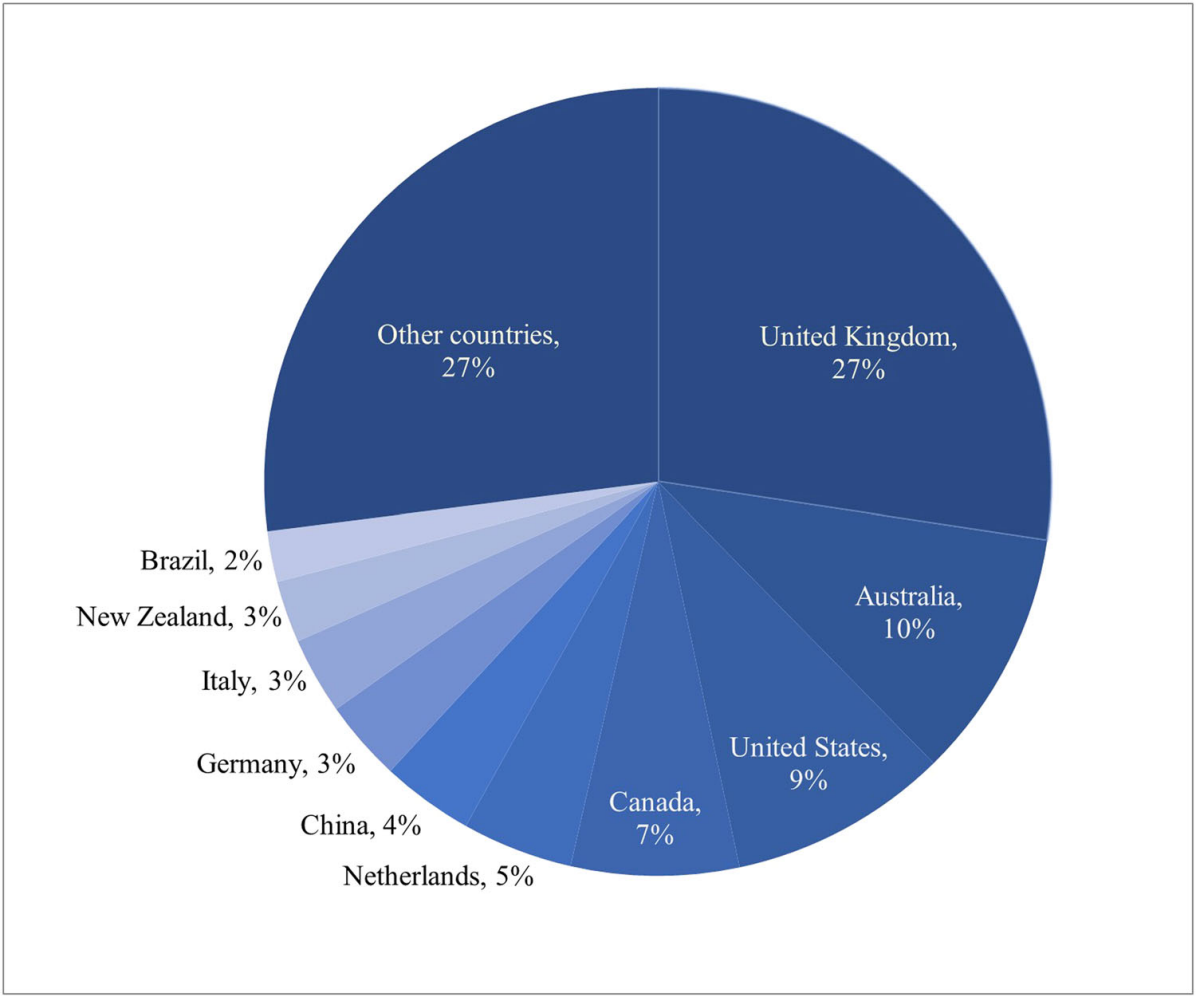
Countries contributing to Cochrane publications indexed in Scopus (1998–2024).

A total of 112 countries actively contributed to the generation of Cochrane evidence during the study period, reflecting a diverse and widespread engagement in evidence synthesis. An analysis of the top 10 countries with the highest number of Cochrane publications showcased a global representation across continents, with notable contributions from various regions. Western Europe demonstrated strong participation, with the United Kingdom (*n* = 5578), Netherlands (*n* = 927), Germany (*n* = 679), and Italy (*n* = 643) emerging as leading contributors. North America was well‐represented by the United States (*n* = 1839) and Canada (*n* = 1403), and Oceania had significant contributions from Australia (*n* = 2091) and New Zealand (*n* = 517). China (*n* = 770) and Brazil (*n* = 424) stood out as the primary representatives of Asia and South America, respectively, among the top 10 countries (Figure 3). Despite the global reach of Cochrane evidence production, countries from other regions did not feature prominently among the top contributors. South Africa made its mark as the first representative of the African continent on the list, ranking 14th with 331 articles, on par with India from Asia.

In terms of impact, the publication rankings were consistent with minor variations. Western Europe was on top of the chart, with the United Kingdom leading in impact (236,908 total citations), followed by the Netherlands (34,744 total citations), Germany (22,513 total citations), Italy (16,701 total citations), and Denmark (15,491 total citations). North America followed, with Canada (59,126 total citations) and the United States (47,029 total citations) making significant contributions. Oceania demonstrated a strong impact through Australia (83,609 total citations) and New Zealand (18,364 total citations). China represented the Asia continent with 14,974 total citations. However, other regions of the world did not feature prominently among the countries with the highest impact, indicating potential areas for growth and collaboration to enhance the global impact of Cochrane evidence.

**Figure 4:**
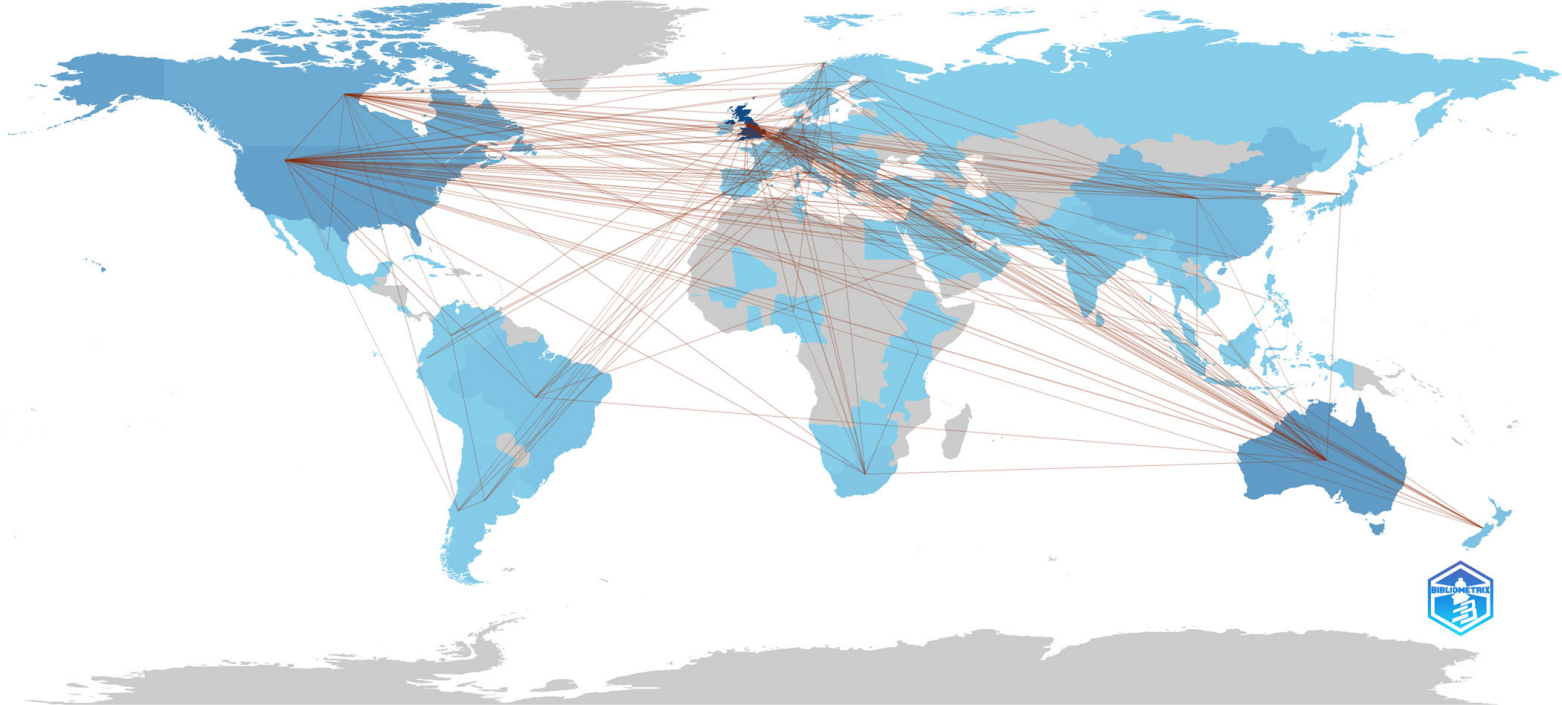
International collaborations in Cochrane systematic reviews indexed in Scopus (1998–2024). Collaborations between countries are shown by red lines. The intensity of colors indicates the volume of publications, with darker shades denoting higher numbers of publications and lighter shades indicating lower numbers. Countries and regions depicted in gray had no available data.

The analysis also revealed extensive international collaborations, with the United Kingdom engaging in significant partnerships with other countries, including Australia (642 collaborations), the United States (559 collaborations), Canada (416 collaborations), and the Netherlands (290 collaborations), showcasing the global reach and collaborative nature of Cochrane systematic reviews. However, the data indicate a lack of representation from countries in Sub‐Saharan Africa, Central Europe, Eastern Europe, and Central Asia (Figure 4).

### 3.4 Prominent funding sources

Analysis of the top funding sources for Cochrane reviews revealed a significant presence of governmental agencies, with 8 out of the top 10 sources emanating from Western Europe, Oceania, and North America. Specifically, significant contributions came from the United Kingdom's National Institute for Health and Care Research (formerly known as the National Institute for Health Research) and the Chief Scientist Office in Scotland. Similarly, the National Health and Medical Research Council in Australia, the Canadian Institutes of Health Research, the National Institutes of Health in the United States, and the National Natural Science Foundation of China also featured prominently. In addition, the World Health Organization, an international organization, and the Eunice Kennedy Shriver National Institute of Child Health and Human Development, a nonprofit organization in the United States, were among the top funding sources. It is worth noting the presence of funding from industrial entities, including Pfizer (*n* = 117), GlaxoSmithKline (*n* = 87), Merck (*n* = 61), Novartis (*n* = 58), and AstraZeneca (*n* = 53) for Cochrane systematic reviews. However, upon closer examination of the primary documents indexed in the Cochrane Library and the National Library of Medicine, these companies were not identified as funding sources for the study. This discrepancy raises concerns about the potential misattribution of funding sources by Scopus.

### 3.5 Keyword analysis

**Figure 5:**
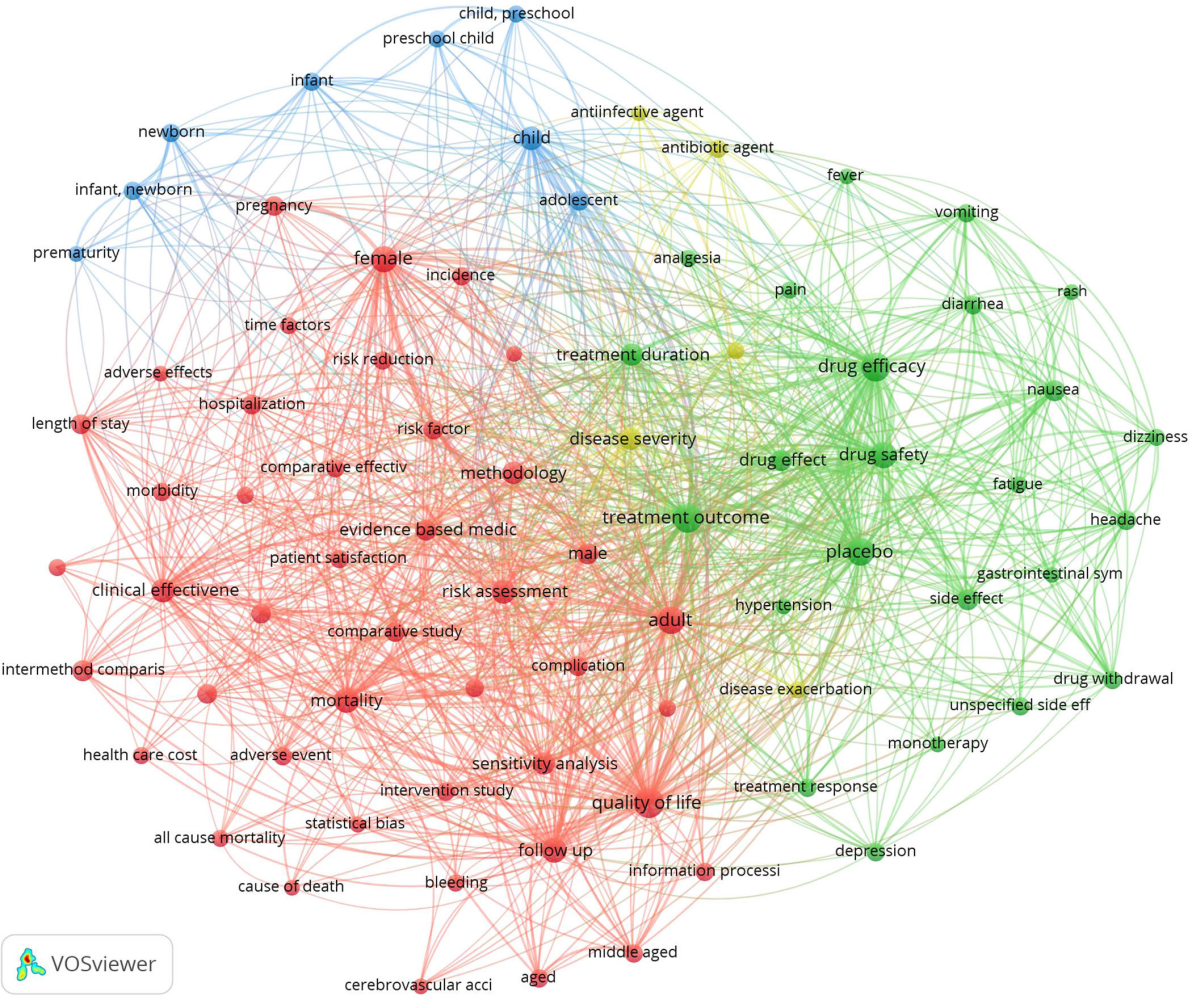
Co‐occurrence network of keywords in Cochrane systematic reviews indexed in Scopus (1998–2024). In this visualization, 80 items form four clusters with 3157 links. Each cluster is color‐coded, with larger nodes indicating higher occurrences in the literature.

In the analysis of both author and index keywords using VOSviewer, a total of 31,301 keywords were identified, with 27,770 attributed to index keywords and 4112 to author keywords. Among these, 8566 keywords were observed to occur at least five times. To enhance the clarity of the network visualization, the threshold for minimum occurrences was adjusted to 100, resulting in the identification of 539 pertinent keywords. Subsequently, the author selected the top 100 keywords for visualization utilizing the association strength method for normalization to enhance the clarity of the diagram. Notably, certain terms such as “Human,” “Review,” “Systematic Review,” “Randomized Controlled Trial,” “Meta‐Analysis,” and “Outcome Assessment” exhibited significantly higher frequencies and the author excluded them from the visualization to streamline the analytical process (Figure 5).

In terms of demographic keywords, “Adult” (*n* = 2522), “Female” (*n* = 2394), “Child” (*n* = 1428), “Male” (*n* = 1091), and “Adolescent” (*n* = 778) emerged as the most frequently occurring terms. When focusing on outcomes, “Quality of Life” (*n* = 3423), “Drug Efficacy” (*n* = 3079), “Treatment Outcome” (*n* = 3212), “Drug Safety” (*n* = 2133), and “Mortality” (*n* = 1960) were among the top five outcomes investigated in Cochrane reviews.

## 4 DISCUSSION

### 4.1 Overview

This study represents the first comprehensive bibliometric analysis of Cochrane reviews spanning from 1998 to 2024, offering intriguing insights into the landscape of Cochrane publications. Results indicate a decline in both Cochrane publications and citations after 2016. Analysis of the contributions from countries, institutions, and authors shows a predominant production of Cochrane evidence by individuals from high‐income nations, possibly attributed to substantial governmental funding and support. Furthermore, the demographic focus of Cochrane evidence leans towards the female sex and adult populations. Subsequent subsections explore identified gaps and propose targeted recommendations. Table 3 summarizes these proposals.

**Table 3:** This table outlines the identified gaps in Cochrane reviews.

Identified gaps	Recommendations	Additional suggestions
1. Underrepresentation of LMIC	Efficiently train LMIC researchers in evidence synthesis.	Specify/create training programs or partnerships with established institutions. Create programs to connect LMIC researchers with experienced researchers from higher‐income regions.
	Involve LMIC experts in existing/upcoming Evidence Synthesis Units and Thematic Groups.	Establish roles for LMIC experts in these groups. Acknowledge their participation on the websites of the groups.
	Provide targeted opportunities for LMIC‐led projects.	Highlight successful LMIC‐led projects as case studies and report them.
	Create incentives for co‐authorship between high‐income and LMIC researchers.	Establish a database of researchers from LMICs to facilitate collaborative opportunities.
2. Limited representation of certain demographics	Focus on geriatric healthcare in existing/upcoming Evidence Synthesis Units and Thematic Groups.	Expand focus to include other marginalized groups, such as individuals with disabilities or indigenous populations.
	Develop/prioritize projects on gender minority health issues.	Incorporate intersectional approaches to address multiple demographic factors.
3. Geographical imbalance	Encourage collaboration among regional Cochrane groups and existing/upcoming Evidence Synthesis Units and Thematic Groups.	Foster international partnerships to enhance research inclusivity.
4. Inefficiencies in evidence production	Streamline evidence synthesis processes, including time‐to‐editorial decisions.	Implement a standardized timeline for manuscript review and publication processes.
	Strengthen partnerships with policymakers to ensure relevance and impact.	Develop workshops/webinars to familiarize policymakers with evidence synthesis processes.
	Prioritize topics of global concern, especially for resource‐limited settings.	Utilize feedback from LMIC stakeholders to identify pressing research topics.
	Familiarize funding sectors with Cochrane's work, particularly in LMICs.	Provide resources and guides to funding bodies on supporting LMIC research initiatives. Inform Evidence Synthesis Units and Thematic Groups about relevant funding opportunities.

*Note*: This table categorizes the gaps into four main areas: underrepresentation of low‐ and middle‐income countries (LMIC), limited representation of certain demographics, geographical imbalances in authorship and evidence production, and inefficiencies in evidence synthesis processes. For each gap, the table presents targeted recommendations and additional suggestions to enhance participation, inclusivity, and effectiveness.

### 4.2 Divergence in search results: Scopus versus Cochrane library

The search in Scopus yielded a substantially higher number of results compared to the Cochrane Library (12,150 vs. 9240 by the time of writing this manuscript). This discrepancy can be attributed to Scopus' inclusion of previous versions of Cochrane reviews. The Cochrane Library search results only include the most recent version of a review and any withdrawn reviews. Cochrane reviews are regularly updated, and a single review may have several previous versions. This iterative process of updating and refining the reviews contributes to the significant difference in the search result counts between Scopus and the Cochrane Library.

### 4.3 Challenges and opportunities in Cochrane's restructuring

While Cochrane has produced a significant number of review articles over the years, the publication and citation patterns have been uneven, with periods of steep decline and fluctuations. The results of the current manuscript suggest a declining trend in both the number of publications and their impact, as measured by citations in recent years.

The creation of Cochrane reviews has been heavily reliant on funding from governmental sectors, which plays a pivotal role in the generation of evidence in different continents. Among the funding sources, the National Institute for Health and Care Research was a major contributor to the development of Cochrane evidence. However, on March 31, 2023, the National Institute for Health and Care Research ceased all core infrastructure funding for Cochrane [4]. This decision led to the closure of several Cochrane groups, particularly the Cochrane review groups based in the United Kingdom, which had been primarily funded through this channel and were the primary source of Cochrane publications. In response to this change, Cochrane has shifted its policy to the establishment of Thematic Groups and Evidence Synthesis Units. These new production models will be responsible for independently securing funding for their operations and will be linked to a Central Editorial Service that will oversee editorial responsibilities. It is crucial that the leaders of these newly developed entities focus on producing high‐quality evidence on priority topics, particularly those applicable to low‐ and middle‐income settings, within a reasonable timeframe. They should avoid unnecessarily prolonging the evidence production process by employing strategies such as rapid systematic reviews or assembling teams of dedicated and passionate individuals with sufficient time to allocate to the synthesis of evidence and ensuring the methodologic rigor. Although this restructuring represents a significant challenge for Cochrane, it presents an opportunity to streamline and optimize the evidence synthesis process. By prioritizing efficiency and quality, Cochrane can continue to fulfill its vital role in providing reliable, up‐to‐date evidence to inform healthcare decision‐making on a global scale.

### 4.4 Enhancing global representation in Cochrane publications

When examining the global landscape of Cochrane publications, the Western European region emerged as a significant contributor, particularly the United Kingdom, owing to established Cochrane centers, robust funding, and research infrastructure. In addition, authorship analysis underscores the diverse collaboration inherent in Cochrane's work, with notable input from Western Europe and Oceania. While this highlights a global effort, the underrepresentation of authors from low‐ and middle‐income countries signals a critical need for increased participation from these regions to enrich the breadth and inclusivity of Cochrane's evidence.

Global health discusses the need for equity and justice in the distribution of healthcare evidence and services worldwide. However, it appears that, as with other assumptions, research that should be helping those living in resource‐limited settings is mainly derived from high‐income countries, and evidence suggests that researchers from low‐resource countries are underrepresented in the publications of journals with high impact [5]. One would argue that individuals living with high standards and access to state‐of‐the‐art technology in healthcare would struggle to comprehend the needs of those with no access to clean water. As a Lancet editorial implicated, global health is a global matter and should not be overwatched by a limited number of people with certain social positions and ethnicities [6]. On the other hand, diversity and inclusivity are cornerstones of Cochrane's policy. The upcoming chapter of Cochrane's agenda should encompass efficient training of the next generation of experts hailing from low‐ and middle‐income countries and those from minority groups in the field of evidence synthesis, so that bright minds could foster international collaborations in the future to enhance the quality of healthcare in these settings. Furthermore, with the formation of Evidence Synthesis Units and Thematic Groups, these groups should highly encourage the inclusion of representative(s) and expert(s) of low‐income and middle‐income countries from various, ethnicities and sexes in their board of directors and ongoing projects. By championing inclusivity through strategic initiatives, Cochrane can further strengthen its impact and relevance on a global scale and serve as a pioneering example for other organizations.

### 4.5 Demographic gaps in cochrane evidence

The keyword analysis provided interesting results. Regarding the demographics indexed in the literature, females were almost twice as represented compared with males. In addition, since most Cochrane evidence was derived from randomized clinical trials and considering that only a small number of the clinical trials include participants from transgender or nonbinary populations [7], the small number of occurrences of such keywords in Cochrane evidence and the current analysis is understandable. The underrepresentation of sexual and gender minority populations in clinical research is a significant concern. Equal access to healthcare is a fundamental human right, and the lack of evidence on the specific needs and experiences of sexual and gender minority populations hinders the provision of inclusive and affirming care. As highlighted by previous studies, there is a pressing need for increased representation and inclusion of sexual and gender minority populations in clinical trials and, subsequently, evidence synthesis [8, 9, 10]. To address this gap, researchers and funding agencies should prioritize the recruitment and retention of diverse participants, including transgender, genderqueer, and nonbinary individuals, in clinical trials, the results of which will subsequently enrich systematic reviews that will have an unwavering impact on the development of clinical guidelines. Tailored recruitment strategies, culturally competent study materials, and partnerships with community organizations can help foster trust and encourage participation. Additionally, the Cochrane evidence should include reports on the gender identity of study participants, using inclusive and respectful terminology, to enable meaningful analyses and comparisons.

### 4.6 Underrepresentation of older adults in Cochrane evidence

Concerns also arise regarding the limited representation of older adults in Cochrane evidence compared to other age groups. Projections indicate a global trend towards declining fertility rates, with estimates suggesting that by 2100, over 95% of countries and territories will experience fertility rates below replacement levels. This demographic shift is expected to result in an aging population and a demographic pyramid inversion, posing significant challenges for nations across various sectors, including national health insurance, social security programs, and healthcare infrastructure [11]. Prioritizing the needs of the geriatric population is crucial and should be a key focus for both existing and upcoming Thematic Groups and Evidence Synthesis Units within Cochrane. By addressing the unique healthcare requirements and challenges faced by older adults, Cochrane can contribute to enhancing the quality of care and support for this growing demographic, ultimately improving health outcomes, and promoting healthy aging globally.

### 4.7 Limitations

This study faced several limitations. Firstly, the abundance of retrieved studies raised the risk of bias due to missing information like authorship and keywords. While efforts were made to ensure data accuracy, the potential for omissions exists. Secondly, the study's broad scope covered publication trends, country participation, authorship, funding sources, and keywords, but word limitations prevented the full presentation of detailed findings. Thirdly, the time‐consuming process of citing recently published articles may have overlooked their impact, authors, and countries in the long term. Additionally, the delayed indexation process in Scopus and the exclusion of authors listed separately posed challenges in real‐time search results and accurate authorship representation. Lastly, the study's reliance on Scopus alone, for practical reasons, may have limited insights compared to databases like Web of Science.

## 5 CONCLUSION

The findings of this study highlight the need for continued exploration and efforts to enhance diversity, funding equity, and inclusivity within Cochrane's evidence‐generation processes to ensure a more comprehensive and representative body of evidence for global healthcare decision‐making, especially from low‐resource settings and minority populations.

## AUTHOR CONTRIBUTIONS

**Amin Sharifan**: Conceptualization; data curation; formal analysis; investigation; methodology; project administration; resources; software; supervision; validation; visualization; writing—original draft; writing—review and editing.

## CONFLICT OF INTEREST STATEMENT

Amin Sharifan has a staff role in Cochrane Austria, is a spoke leader for the Cochrane Planetary Health Thematic Group, is an associate editor of Cochrane Clinical Answers, is a steering member of the Cochrane Early Career Professionals Network, was a previous mentee of the Cochrane US Mentoring Program, was a member of Cochrane's Evidence Synthesis Unit Assessment Panel, and reports voluntary collaborations with Cochrane Sweden, Cochrane Canada, Cochrane Hepato‐Biliary Group, Cochrane First Aid, and Cochrane Heart, Stroke and Circulation Thematic Group.

## ETHICS STATEMENT

This study was carried out without the involvement of human participants or animal subjects. Consequently, due to the reviewing nature of the research, ethical approval was not necessary.

## Data Availability

Raw data were generated from Scopus database. Derived data supporting the findings of this study are available to bona fide researchers from the corresponding author AS on reasonable request.
